# Repair of olecranon fractures using fiberWire without metallic implants: report of two cases

**DOI:** 10.1186/1749-799X-5-73

**Published:** 2010-10-12

**Authors:** Akimoto Nimura, Teruhiko Nakagawa, Yoshiaki Wakabayashi, Ichiro Sekiya, Atsushi Okawa, Takeshi Muneta

**Affiliations:** 1 Section of Orthopedic Surgery, Graduate School, Tokyo Medical and Dental University, 1-5-45 Yushima, Bunkyo-ku, Tokyo, 113-8519 Japan; 2 Department of Orthopedic Surgery, Doai Memorial Hospital, 2-1-11 Yokoami, Sumida-ku, Tokyo, 130-8587 Japan; 3 Section of Cartilage Regeneration, Graduate School, Tokyo Medical and Dental University, 1-5-45 Yushima, Bunkyo-ku, Tokyo, 113-8519 Japan

## Abstract

Olecranon fractures are a common injury in fractures. The tension band technique for olecranon fractures yields good clinical outcomes; however, it is associated with significant complications. In many patients, implants irritate overlying soft tissues and cause pain. This is mostly due to protrusion of the proximal ends of the K-wires or by the twisted knots of the metal wire tension band. Below we described 2 cases of olecranon fractures treated with a unique technique using FiberWire without any metallic implants. Technically, the fragment was reduced, and two K-wires were inserted from the dorsal cortex of the distal segment to the tip of the olecranon. K-wire was exchanged for a suture retriever, and 2 strands of FiberWire were retrieved twice. Each of the two FiberWires was manually tensioned and knotted on the posterior surface of the olecranon. Bony unions could be achieved, and patients had no complaint of pain and skin irritation. There was only a small loss of flexion and extension in comparison with that of the contralateral side, and the patient did not feel inconvenienced in his daily life. Using the method described, difficulty due to K-wire or other metallic implants was avoided.

## Background

Olecranon fractures consist of approximately 10% of all fractures around the elbow. The tension band fixation is the commonest technique for relatively simple fractures. This technique combines intramedullary Kirshner wires (K-wires) with a metal wire tension band. The AO tension band technique yields good clinical outcomes; however, it is associated with significant complications[1-3]. In many patients, implants irritate overlying soft tissues and cause pain. This is mostly caused by protrusion of the proximal ends of the K-wires or by the metal wire tension band. It may be necessary to remove the implant, occasionally before fracture union. It is clearly desirable to find a fixation method that enables surgeons to rigidly fix the fracture site without skin irritation related to the backing out of hardware. The purpose of this case report is to introduce our unique method for olecranon fractures using high-strength suture without any metallic implants.

## Case presentation

### Case 1

**Figure 1:**
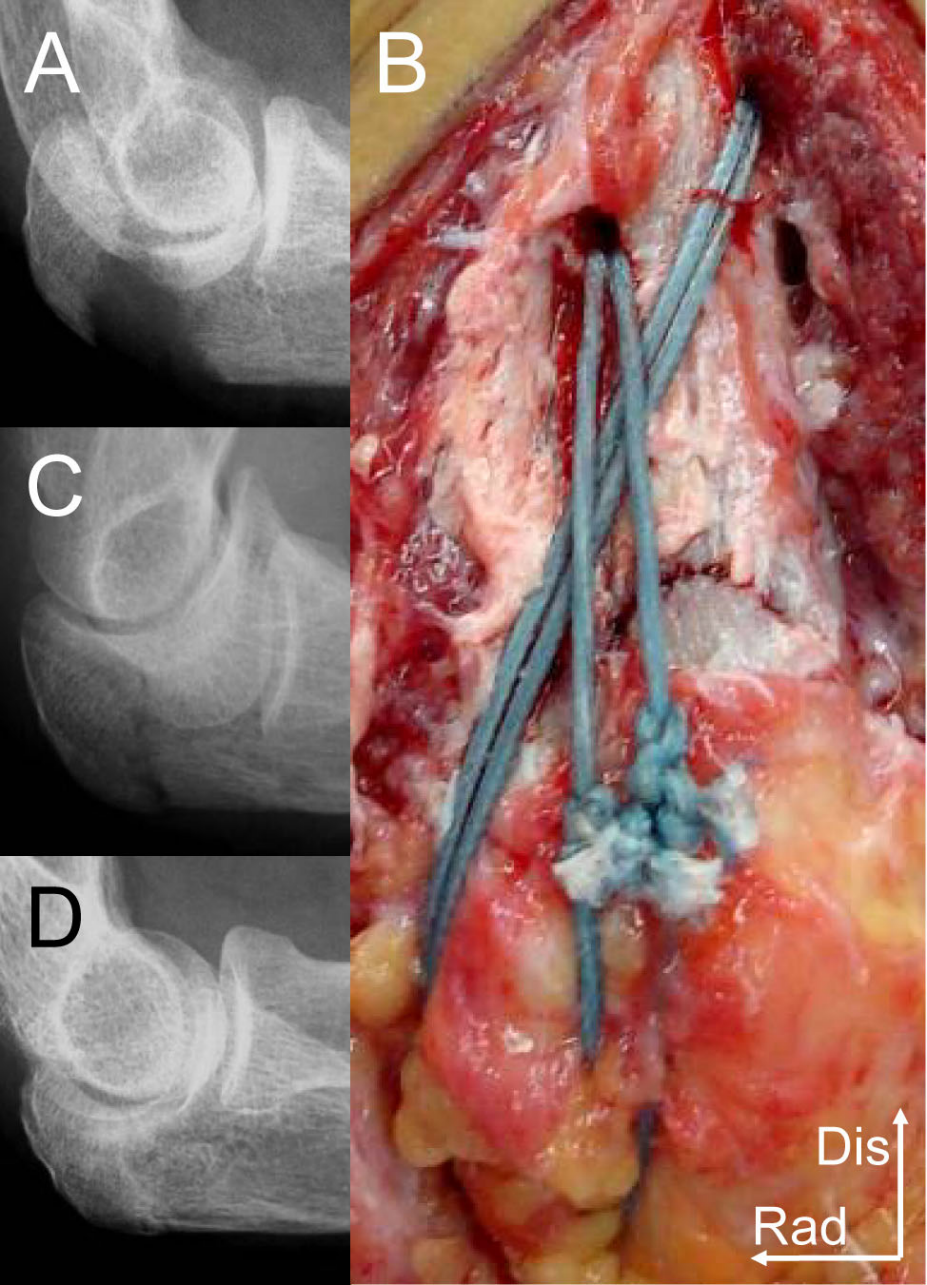
**Radiographs and the intraoperative photograph of case 1**. (A) Lateral radiograph of preoperative period. (B) The fracture site after fixation with 2 strands of FiberWire. Dis; distal. Rad; radial. (C) Lateral radiograph of intraoperative period. (D) Radiograph of 1 year postoperative period.

A 56-year-old, right-dominant woman fell on her left elbow after an accident riding a bicycle. Two days after injury, she was admitted to our hospital. Radiography revealed an olecranon fracture, which was classified into type II-A by Mayo classification[4] (Figure 1A). Surgery was carried out 6 days after injury.

**Figure 2:**
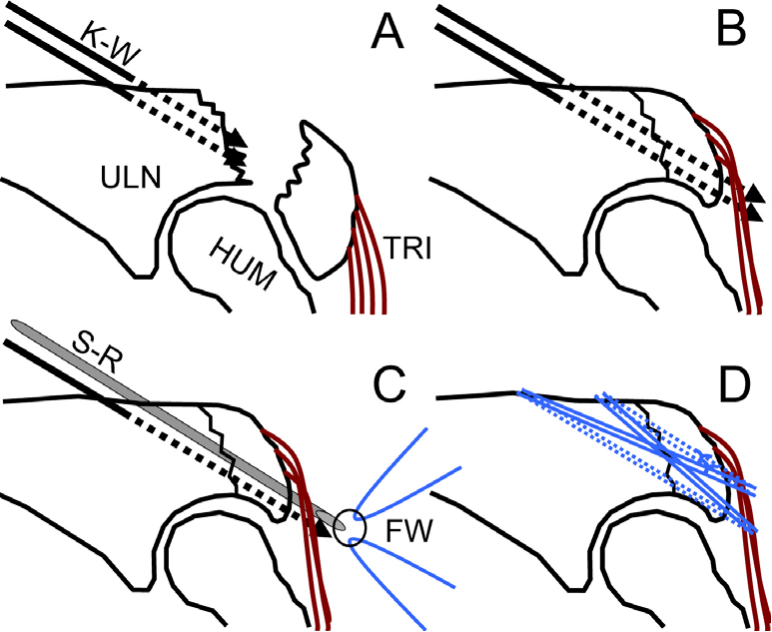
**Surgical techniques using FiberWire**. (A) Two K-wires were passed. HUM; Humerus. K-W; Kirshner wire. TRI; Triceps tendon. ULN; Ulna. (B) After resetting the segment, K-Wires were placed across the fracture site. (C) Two strands of FiberWire were retrieved with Suture-Retriever. FW; FiberWire. S-R; Suture Retriever. (D) Two sutures were knotted on the olecranon.

The operation was performed with the patient in the supine position and the arm over the chest under regional anesthesia with an axillary block and under tourniquet control. By use of a posterior midline skin incision on the tip of the olecranon, the fracture was exposed. Two 2.0 mm K-wires were passed from the fracture site of distal segment to dorsal cortex parallel. The two K-wires were reversely directed using the same hole from dorsal cortex to the fracture site (Figure 2A). The fragment was reduced, and the two K-wires were inserted from the dorsal cortex of the distal segment to the tip of the olecranon (Figure 2B). One of the K-wires was exchanged into a suture retriever (Smith and Nephew, Memphis, TN) using the same hole (Figure 2C). Two strands of No. 5 FiberWire (Arthrex, Naples, FL) were retrieved twice in the same fashion. Each of the two No. 5 sutures was tensioned manually and knotted on the posterior surface of the olecranon (Figure 1B, C, 2D).

Postoperatively, the elbow was immobilized with a plaster splint for 2 weeks. At one year after the surgery, bony union still had been achieved (Figure 1D). The patient had no complaint of pain and skin irritation. Range of motion at this time was 0°-15°-145° in flexion-extension. The patient did not feel inconvenienced in her daily life. She scored 11.6 on the postoperative DASH score (the JSSH version) at one year of follow up[5].

### Case 2

**Figure 3:**
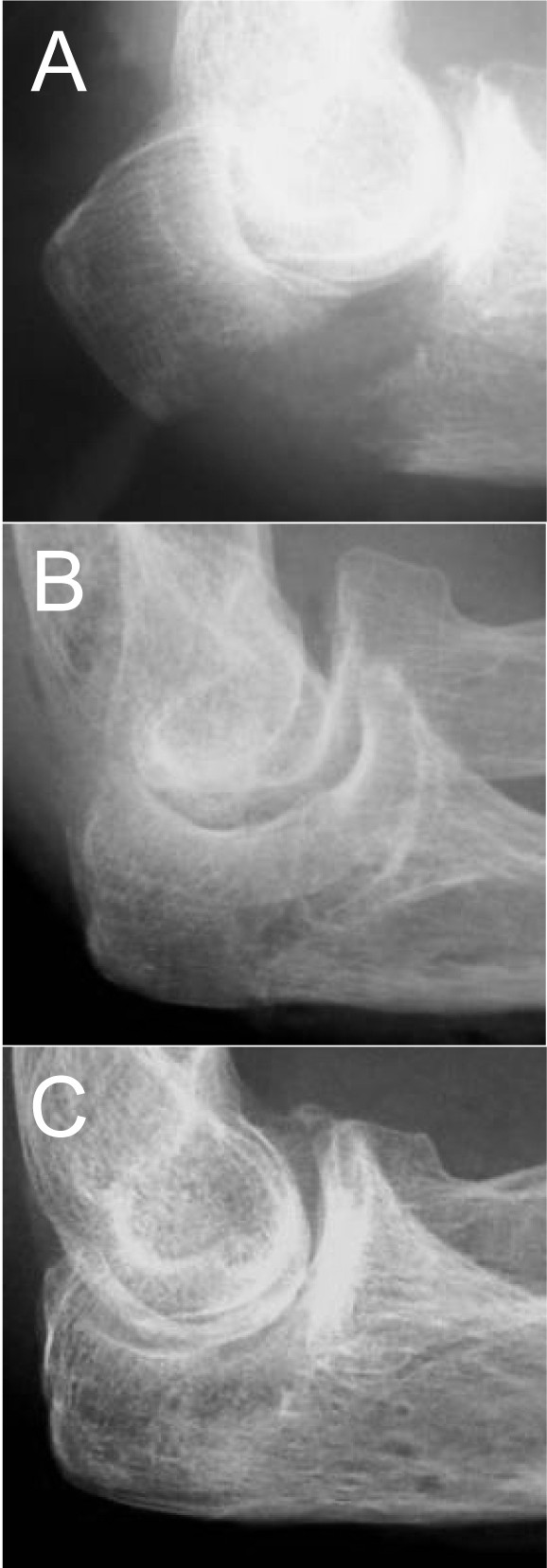
**Radiographs of case 2**. (A) Lateral radiograph of preoperative period. (B) Lateral radiograph of intraoperative period. (C) Radiograph of 1 year postoperative period.

The next patient was an 84-year-old right dominant woman who fell on her right elbow. She was immediately admitted to our hospital. Radiography revealed an olecranon fracture, which was classified into type II-A by Mayo classification (Figure 3A). Surgery was carried out 9 days after injury.

The operation was performed under general anesthesia. The fracture site was fixed with two strands of No. 5 FiberWire using the same technique at that of case 1 (Figure 3B).

Postoperatively, the elbow was immobilized with a plaster splint for 2 weeks. At one year after the surgery, bony union was still achieved (Figure 3C). The patient had no complaint of pain and skin irritation. Range of motion at this time was 0°-15°-145° in flexion-extension. The patient did not feel inconvenienced in her daily life. She scored 12.1 on the postoperative DASH score (the JSSH version) at one year of follow up.

## Conclusion

Olecranon fractures are a common injury in fractures. In general, displaced fractures are treated by open reduction and internal fixation. Several fixation methods have been described in the literature including the tension band technique[4], intramedullary screws[6], and plate fixation[7]. The AO tension band is appropriate for non-comminuted fractures. It is believed that the tension band converts the distractive forces generated by the triceps at the posterior surface into compressive forces at the anterior articular surface.

Although tension band wiring is a widely accepted technique for olecranon fracture fixation with good reported long-term results, numerous postoperative problems have been reported[1-3]. In many patients, implants irritate overlying soft tissues and cause pain. This is mostly caused by protrusion of the proximal ends of the K-wires or by the metal wire tension band, particularly its twisted knots. Macko et al.[1] described that in 20 patients treated with tension band wiring, 16 experienced symptomatic prominence of the K-wires and 4 experienced skin breakdown. Helm et al.[2] reported that 82% of their patients needed hardware removal following tension band wiring. Specific problems are related to the subcutaneous position of the K-wires and knots of metal wire; whose migration may be responsible for secondary fracture displacement, soft-tissue problems, and local pain. It may also be necessary to remove the implant, occasionally before fracture union. Despite these reported problems, tension band wiring in displaced olecranon fractures is still the gold standard. It is clearly desirable to find a fixation method that enables surgeons to fix the fracture site without skin irritation related to the backing out of hardware.

Previously, Carofino et al.[8] reported tension band constructed with FiberWire when used with either an intramedullary screw or K-wire provide fixation of olecranon fractures equivalent to an 18-gauge metal wire in order to reduce the incidence of skin irritation. This technique was a new idea, but it is not able to prevent irritation with screw heads or K-wire ends when they back out, because these methods use metallic screws and K-wires which is the same as that of traditional tension band. In the present report, we developed an innovative technique with which olecranon fractures could be fixed with FiberWire without any metallic implants and prominence of hardware, and subcutaneous irritation could thus be avoided. Additionally, because these problems oriented to K-wire or to other metallic implants should be prevented, second operations of hardware removal would not be necessary.

FiberWire is high-strength braded suture composed of polyester and polyethylene and has over twice the strength of traditional suture. Wust et al.[9] reported that the ultimate strength of FiberWire was 2-to 2.5-fold greater than that of polyester or polydioxanone sutures. FiberWire has been used in place of metal wire in other orthopaedic applications without loss of strength. Wright et al. presented that double-strand FiberWire had a significantly higher failure load than stainless steel wire, when they were used for tension band fixation on a novel transverse patellar fracture model and tested to failure by three-pointing bending[10].

Based on our experiences, the stabilities of fracture accompanied with comminutions on the distal part of the fracture site could not be obtained using the novel technique. This seems to be the limitation of this technique. Though we have not tried yet, the present method could be applied to fixations after osteotomy of the olecranon thus preventing irritation caused by metallic implants.

We demonstrated a unique technique for olecranon fractures using only high-strength suture. Using the method described, the obstacles which include soft-tissue problems, local pain, and hardware removal related to the use of K-wire or other metallic implants could be avoided. Our fixation technique for olecranon fractures using FiberWire without metallic implants could be an alternative treatment for tension band wiring.

## Consent

Written informed consent of case 1 was obtained from the patient, herself and that of case 2 was obtained from the patient's relatives for publication of this case report.

## Competing interests

The authors declare that they have no competing interests.

## Authors' contributions

AN, who is the corresponding author, has contributed in conception and design and acquisition of data, analysis and interpretation of data, drafting the manuscript and revising it critically. TN has contributed in acquisition of data and revising the manuscript. YW has contributed in conception and design of data and revising the manuscript. IS has contributed in revising the manuscript. AO and TM has contributed in final approval of manuscript. All authors have read and approved the final manuscript.
